# Institutional Questions

**Published:** 1900-07-28

**Authors:** 


### INSTITUTIONAL QUESTIONS.

### Rating of Hospitals.

"Honorary Secretary" writes: Will you oblige me by advice upon this point? Our hospital, which has just been opened, and which has cost in building alone about £4,000, the land having been given, has just been assessed to the poor rate on a net basis of £66. That seems to us a very large sum, especially as another hospital not far away which is equally large is only assessed at £36. Could you tell me what is the rule followed in the assessment of hospitals? Our accommodation is twelve beds in two small wards.

*** We would advise you to approach the local rating authority and point out (1) that the hospital is of material assistance to the rates by taking in cases which must relieve the poor law; (2) for this reason the more intelligent rating authorities throughout the country, taking this point into consideration, have decided to levy rates upon that portion of the hospital buildings only which are beneficially occupied, *i.e.*, the parts in which the paid officials reside. In this way we should think you ought to get your rating reduced by two-thirds to three-quarters. You are of course aware that a Parliamentary Committee has just reported in favour of relieving hospitals from the payment of rates.

### Heating and Ventilation.

"A. B. C." asks: I am at present engaged with plans for a new hospital to contain 60 beds. With regard to the heating arrangements, would you kindly tell me who is the best practical man to employ, and also whether it would be best to (1) Heat the institution throughout with fireplaces and hot-water pipes in all the wards; or (2) heat with fireplaces in the wards and hot-water pipes only in the corridors; or (3) heat entirely with hot-water pipes and have no fireplaces in the wards? The latter plan would be a great saving in many ways, but I do not know whether it is advisable or not.

*** You should consult a competent architect as to the "best practical man"; no one else can properly advise you. As a first step, you should get an excellent little paper on "The Sanitation of Hospitals and Infirmaries," by Mr. Keith D. Young, F.R.I.B.A., printed by Wyman and Sons, 74, Great Queen Street, Lincoln's Inn Fields, W.C. Mr. Young is responsible for some admirable modern hospitals, two of those most recently built being the Royal London Ophthalmic Hospital, City Road, E.C., and the new buildings of the Portsmouth Royal Hospital. A visit to these would show you the system which, in our view, is the most satisfactory for hospital buildings, namely, heating by means of open fireplaces and by hot coils, or radiators, combined. For many reasons the absence of open fires is objectionable, not the least of these being that a bright, open fire has a cheerful, moral effect in a ward or sick room, as in any living room, and that it is far easier to maintain a sweet, clean atmosphere with open grates than where heating is carried out by solely artificial means, with all its consequent complications of machinery and working.

